# Description of the interaction between *Candida albicans* and macrophages by mixed and quantitative proteome analysis without isolation

**DOI:** 10.1186/s13568-015-0127-2

**Published:** 2015-07-16

**Authors:** Nao Kitahara, Hironobu Morisaka, Wataru Aoki, Yumiko Takeda, Seiji Shibasaki, Kouichi Kuroda, Mitsuyoshi Ueda

**Affiliations:** Division of Applied Life Sciences, Graduate School of Agriculture, Kyoto University, Sakyo-ku, Kyoto, 606-8502 Japan; Japan Society for the Promotion of Science, Sakyo-ku, Kyoto, 606-8502 Japan; General Education Center, Hyogo University of Health Sciences, Chuo-ku, Kobe, 650-8530 Japan; Division of Rheumatology, Department of Internal Medicine, Hyogo College of Medicine, Nishinomiya, Hyogo 663-8501 Japan

**Keywords:** *Candida albicans*, Macrophage, Mixed proteome analysis, Quantitative proteome analysis, Apoptosis, Chaperone

## Abstract

*Candida albicans* is an opportunistic pathogen that causes fatal diseases in immunocompromised hosts. Host resistance against *C. albicans* relies on ingestion of the pathogen by macrophages. Analysis of the escaping behavior of *C. albicans* from macrophages is required to understand the onset of systemic candidiasis. In this study, native interactions of *C. albicans* with macrophages were investigated by proteome analysis using high efficiency of long monolithic silica capillary column. Using this system, we developed a method of “mixed and quantitative proteome analysis” in which *C. albicans* and macrophages were simultaneously analyzed by nanoLC–MS/MS without the need to isolate the two individual living cells. Two hundred twenty-seven proteins from *C. albicans* and five proteins from macrophages were identified as candidate interaction-specific molecules. *C. albicans* seemed to produce glucose through a β-oxidation pathway, a glyoxylate cycle, and gluconeogenesis for escape from macrophages. Up-regulation of stress-related and candidate pathogenic proteins in *C. albicans* indicated how *C. albicans* endured the harsh environment inside the macrophages. Down-regulation of apoptosis-associated protein NOA1- and chaperone HSPA1A-syntheses in macrophage indicated that *C. albican*s was able to escape from macrophages in part by suppressing the production of these macrophage proteins.

**Electronic supplementary material:**

The online version of this article (doi:10.1186/s13568-015-0127-2) contains supplementary material, which is available to authorized users.

## Introduction

*Candida* species usually reside as commensal fungi of humans as part of the normal microflora of skin, the oral cavity, the gastrointestinal tract, and the vagina (Scanlan and Marchesi 2008; Standaert-Vitse et al. 2009). However, *Candida* species behave as aggressive pathogens in immunosuppressed individuals such as HIV patients and cancer patients who are undergoing chemotherapy (Luo et al. 2013). In systemic candidiasis, the dissemination of *C. albicans* begins with invasion into mucosal surfaces and subsequent entries into the bloodstream (Szabo and MacCallum 2011). Thereafter, *C. albicans* shows hematogenous spread to virtually any organs, including the brain, kidney, liver, and lung, often leading to the patient death (Koh 2013). The mortality rate of systemic candidiasis is as high as 50% because of the lack of effective diagnostics and treatments (Mayer et al. 2013). Hence, there is an urgent need to elucidate virulence mechanisms of *C. albicans* to support the development of effective drugs.

The innate immune system, in particular macrophages, is the first step of host defenses against pathogenic fungi (Miramon et al. 2013). Macrophages are able to kill microorganisms by phagocytosis and attract other immune cells by producing cytokines (Galli et al. 2011). However, following phagocytosis, *C. albicans* tears and kills macrophages, eventually escaping from them (Seider et al. 2010). Little is known about the mechanisms used by *C. albicans* to escape from macrophages (Vazquez-Torres and Balish 1997). Many bacterial proteins have been identified as pathogenic effectors for killing macrophages, such as *Salmonella* SipA (Lilic et al. 2003) and PipB2 (Henry et al. 2006) that bind macrophage actin and kinesin, respectively. These interactions give *Salmonella* the ability to migrate within the macrophage, whereas *Salmonella* SipB binds to macrophage caspase-1 and causes macrophage pyroptotic cell death (Hersh et al. 1999).

The purpose of this study is to understand the comprehensive proteome responses occurring during natural interactions between *C. albicans* and macrophages using a quantitative proteome analysis. For this proteome analysis, we applied a monolithic silica capillary column to separate peptides in a liquid chromatography (LC) step. The monolithic silica capillary column is made of silica skeletons and through-pores (Kobayashi et al. 2006). This unique structure gives monolithic column with large flow-through pores. This allows for a higher separation efficiency and lower-pressure drop than conventional particle-packed columns (Motokawa et al. 2002). Recently, a system using a long monolithic silica capillary column successfully identified 2,602 proteins produced in *Escherichia coli* cells in a single analysis (Iwasaki et al. 2010). Using the high separation efficiency of the monolithic column, we tested its ability to support a mixed and quantitative proteome analysis.

**Figure 1:**
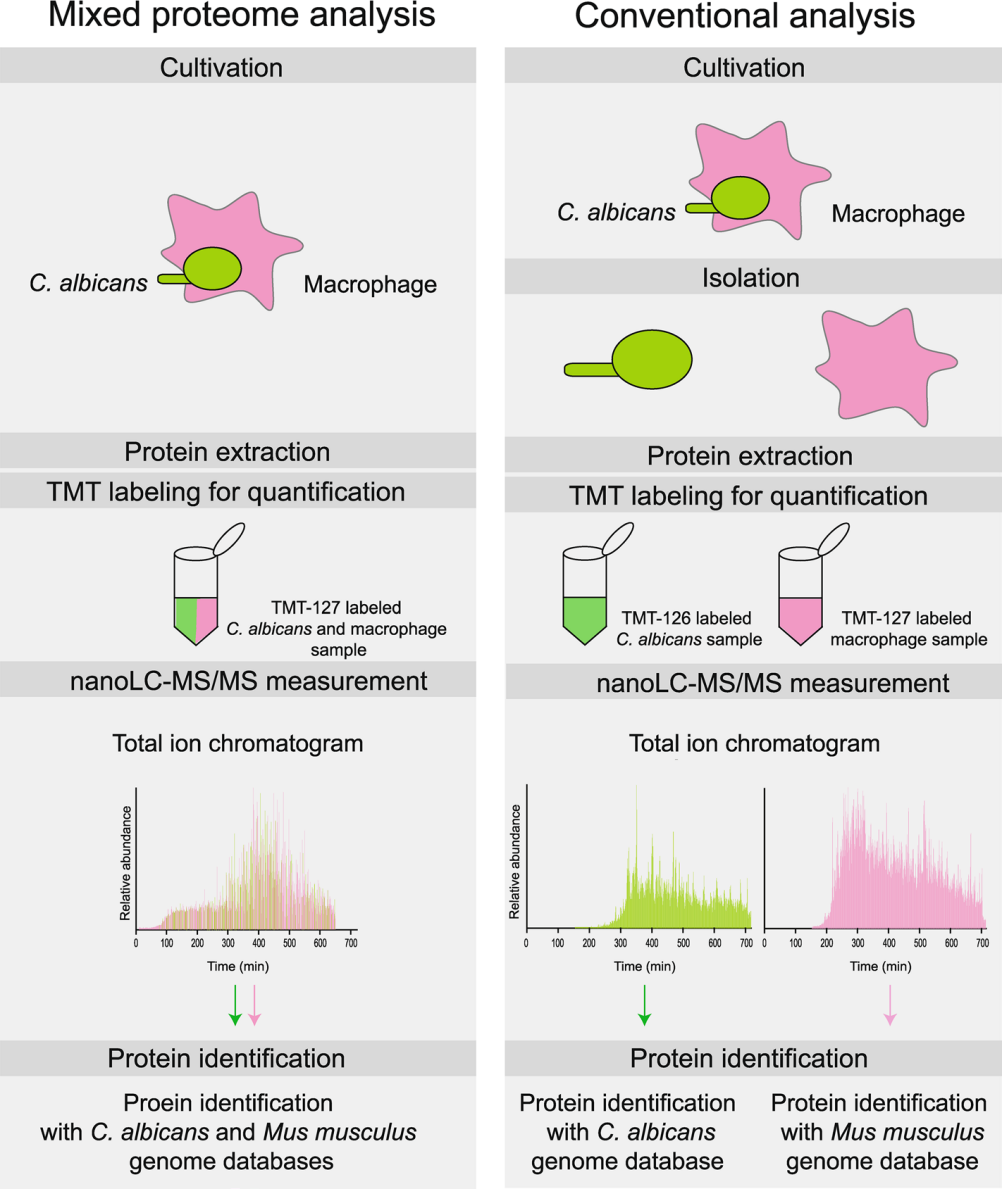
Method of mixed and quantitative proteome analysis, in which protein production in two organisms can be measured without their isolation.

In the mixed and quantitative proteome analysis, samples prepared from *C. albicans* and macrophages were directly analyzed by nanoLC–MS/MS without isolating the individual *C. albicans* and macrophage cells in the co-culture (Figure 1). Using genome data, we could distinguish each peptides derived from the individual organisms by referring individual genome sequences. Omitting the individual cell isolation steps is important because these steps are known to alter the natural states of protein networks by causing various artifacts from unnecessary stresses (Reales-Calderon et al. 2012; Rupp 2004; Fernandez-Arenas et al. 2007; Reales-Calderon et al. 2013). This is the first report of a mixed and quantitative proteome analysis in which the purification and fractionation processes were completely omitted.

We identified a total of 483 *C. albicans* proteins and 1,253 macrophage proteins. Furthermore, we identified 227 *C. albicans* and five macrophage proteins with altered production level. Macrophage-induced *C. albicans* proteins were associated with glucose generation, membrane synthesis, stress response, and other unknown functions. *C. albicans*-induced macrophage proteins were associated with apoptosis and a chaperone. The escape of *C. albicans* from macrophages could be apparently mediated by the production of these *C. albicans* proteins and the degradation of macrophage proteins.

## Materials and methods

### Strain

*Candida albicans* strain SC5314/ATCC® MYA-2876™ (American Type Culture Collection, Manassas, VA, USA) was maintained on yeast extract-peptone-dextrose (YPD) medium (1% *w/v* yeast extract, 2% *w/v* glucose, and 2% *w/v* peptone) and incubated at 30°C. J774.1 murine macrophages (RIKEN BioResource Center, Ibaragi, Japan) were maintained in complete culture medium [DMEM media containing 10% fetal bovine serum (FBS), 100 units/ml penicillin, and 100 μg/ml streptomycin (Life Technologies, Carlsbad, CA, USA)] at 37°C in a humidified atmosphere with 5.0% CO_2_. Cells were maintained at low densities (75% confluence) and passaged until reaching the confluent state, usually every 3–4 days on 90 mm cell culture dishes. For phagocytic experiments, macrophage cells were plated at 1.0 × 10^5^ cell/well for 16 h prior to the experiments.

### Interaction of macrophages with *C. albicans*

A total of 5.0 × 10^6^ macrophage cells were plated in complete culture media in culture dishes for 16 h prior to the experiments. *C. albicans* was pre-cultivated in 10 ml YPD media for 12 h. These *C. albicans* cells were washed with complete culture medium, counted with a hemocytometer and diluted to 5.0 × 10^6^ cell/ml in 50 ml of complete culture medium. A total of 5.0 × 10^6^*C. albicans* cells were added per macrophage dish to obtain a fungus-macrophage ratio of 10:1 and incubated for the indicated times at 37°C and under 5.0% CO_2_.

### Measurement of tumor necrosis factor-α (TNF-α)

Fifty microliters of the supernatant of *C. albicans*-macrophage interaction cultures were collected at 0, 1, 2, 3, 4, 5, 6, 7, 8, 9, 12, and 24 h to determine TNF-α levels. The amount of TNF-α was determined using a mouse TNF-α ELISA kit (R&D systems, Minneapolis, MN, USA), according to the manufacturer’s instructions.

### Isolation of proteins

The macrophage cells were incubated with *C. albicans* cells for 3 h for proteome analysis. After three washing with ice-cold phosphate-buffered saline (PBS; pH 7.4, 1.4 M NaCl, 81 mM Na_2_HPO_4_, 27 mM KCl, 15 mM KH_2_PO_4_), the macrophages and *C. albicans* cells were dislodged by scraping the dish with rubber scrapers in ice-cold wash buffer [20 mM Tris-HCl, pH 7.8, containing 1.0% protease inhibitor cocktail for use with mammalian cell and yeast extract (Life Technologies)]. Collected samples were frozen quickly using liquid nitrogen and preserved at −80°C until use. Proteins were extracted as described previously with minor modifications (Aoki et al. 2013). Briefly, each sample was centrifuged for 5 min at 3,000×*g*, and the resulting cell pellets were suspended in 400 μl of lysis buffer [4% *w/v* 3-(3-cholamidepropyl)dimethylammonio-1-propanesulfonate, 1% *w*/*v* dithiothreitol, 1% *v*/*v* protease inhibitor cocktail for mammalian and yeast cells, 7 M urea, and 2 M thiourea in 20 mM Tris-HCl, pH 7.8]. The solution was mixed with 200 mg of 0.5 mm beads (TOMY SEIKO, Tokyo, Japan), and the cells were mechanically disrupted 10 times using a BeadSmash 12 (Wakenyaku, Kyoto, Japan) at 4°C, 4,000 oscillations per minute for 1 min. The solution was centrifuged at 3,000×*g* for 15 min and the supernatant was collected. Two hundred microliters of 200 mM triethyl ammonium bicarbonate (TEAB; Sigma-Aldrich, St. Louis, MO, USA) was added to each pellet and centrifuged at 3,000×*g* for 15 min. The supernatant was combined with the previously collected supernatant and the solutions were concentrated using Amicon Ultra YM-10 (Millipore, Bedford, MA, USA) with buffer exchange into 200 mM TEAB. The concentrated samples were dissolved in 100 μl of 200 mM TEAB.

### Reduction, alkylation, and digestion

The sample solutions were mixed with 135 µl of 200 mM TEAB and 30 μl of 200 mM tris-(2-carboxyethyl) phosphine. The solutions were incubated at 55°C for 1 h for reduction. After the reaction, 60 μl of 375 mM iodoacetamide was added to the solutions and incubation was continued for 30 min at room temperature, with protection from light. The reactants were mixed with 1 ml of ice-cold acetone and incubated at −20°C for 2 h to precipitate the proteins. The precipitated proteins were suspended with 250 μl of 200 mM TEAB and mixed with 2 μl of sequencing grade modified trypsin (1 µg/μl) (Promega, Fitchburg, WI, USA). The mixture was incubated at 37°C for 12 h. Peptide concentration was determined using the Bicinchoninic acid assay kit (Nacalai Tesque, Kyoto, Japan) according to the manufacturer’s instructions.

### TMT labeling

The peptide solutions were labeled using the TMT sixplex Isobaric Label Reagent Set (Thermo Fisher Scientific, Waltham, MA, USA), according to the manufacturer’s protocol. The TMT-labeling reagents were dissolved in 41 µl acetonitrile and mixed with 0.75 µg of each digest. In brief, a total of 0.75 µg of the proteins from the monoculture and co-culture were mixed with TMT-126 and -127, respectively. In addition, an equal amount of a mixture containing all of the samples types was labeled with TMT-131 as an internal control for quantification. The reactions were quenched by the addition of 8 µl of 5% hydroxylamine, followed by combining and lyophilizing the solutions. The dried samples were dissolved in 200 µl of 0.1% formic acid for calibration of TMT labeling. To measure the precision of protein quantification with the nanoLC–MS/MS system, the standard sample was separated into three tubes at a ratio of 0.5:1:2 by volume and the samples were labeled with TMT-128, TMT-130, and TMT-131, respectively. After quenching the reaction, these samples were combined in a single tube and injected into the nanoLC–MS/MS and the relative intensities of reporter ions of each identified protein were calculated.

### LC–MS/MS measurement

Proteome analyses were performed using a nanoLC (Ultimate 3000®; Thermo Fisher Scientific)-MS/MS (LTQ Velos orbitrap mass spectrometer®; Thermo Fisher Scientific) system. Tryptic digests were injected and separated by reversed-phase chromatography using a long monolithic silica capillary column, which was prepared from a mixture of tetramethoxysilane and methyltrimethoxysilane (500-cm long, 0.1-mm ID) as described previously (Motokawa et al. 2002; Aoki et al. 2013), at a flow rate of 500 nl min^−1^. A gradient was established by changing the mixing ratio of the two eluents; A, 0.1% (*v/v*) formic acid; and B, 80% acetonitrile containing 0.1% (*v/v*) formic acid. The gradient was started with 5% B, increased to 45% B for 600 min, further increased to 95% B to wash the column and then returned to the initial condition and held for re-equilibration. The separated peptides were detected on the MS with a full-scan range of 350–1,500 *m/z* (resolution 60,000) in the positive mode followed by 10 data-dependent high-energy C-trap dissociation (HCD) MS/MS scans to acquire TMT reporter ions. For data-dependent acquisition, the method was set to automatically analyze the top 10 most intense ions observed in the MS scan. An ESI voltage of 2.4 kV was applied directly to the LC eluent distal to the chromatography column. Normalized collision energy of 40% in HCD with 0.1 ms activation time was used. The dynamic time exclusion was 180 s. The ion-transfer tube temperature on the LTQ Velos ion trap was set to 300°C. Triplicate analyses were done for each sample of four biological replicates.

### Data analysis

The mass spectrometry data of each biological replicate was used for protein identification and quantification. Analysis was performed using Proteome Discoverer 1.2 (Thermo Fisher Scientific). Protein identification was performed using MASCOT (Matrix Science, London UK, USA) against the Assembly 21 *Candida* genome database (6,198 sequences) for *C. albicans* and against the *Mus musculus* database (25,530 sequences) from the common part of NCBI (http://www.ncbi.nlm.nih.gov/) and IPI (http://www.webcitation.org/getfile?fileid=ccad550bc21e5bcf0f4b8763a56240fcb7058693) database with a precursor mass tolerance of 50 ppm, a fragment ion mass tolerance of 20 mmu and strict specificity allowing for up to one missed cleavage. For trypsin digestion, carbamidomethylation of cysteine, TMT sixplex of N-term (+229.1629 Da) and TMT sixplex of lysine (+229.1629 Da) were set as fixed modifications. The data were then filtered at a *q* value ≤0.01 corresponding to a 1% false discovery rate (FDR) on a spectral level. Protein quantification was performed by Reporter Ions Quantifier with the TMT sixplex method on Protein Discoverer. Four independent biological experiments were performed and proteins identified in every replicate were considered.

### Calculation of false positive proteins rates

*Candida albicans* monoculture, macrophage monoculture, and complete culture medium were incubated for 3 h and the proteins as a control were extracted, reduced, alkylated, and digested with trypsin. Tryptic digests were analyzed by nanoLC–MS/MS system with a long monolithic silica capillary column under the same conditions. Triplicate analyses were performed for each sample of three biological replicates. The mass spectrometry data were used for protein identification using MASCOT, working on Proteome Discoverer with a peptide tolerance of 1.2 Da, MS/MS tolerance of 0.8 Da, and maximum number of missed cleavages of two. For trypsin digestion, cysteine carbamidomethylation (+57.021 Da) and methionine oxidation (+15.995 Da) were set as a variable modification. The data were then filtered at a *q* value <0.01 corresponding to 1% false discovery rate on a spectral level. To identify false positive proteins, proteins derived from each organism and complete culture medium were analyzed with *C. albicans*, *M. musculus* database for macrophage, and *Bos taurus* database for complete culture medium from NCBI (http://www.ncbi.nlm.nih.gov/genome?term=bos%20taurus). Proteins identified by detection of at least two peptides in any of three biological replicates or by a single peptide at all three biological replicates were considered as ‘false positive protein’ and are listed in Additional file 1.

### Extraction of differentially produced proteins

After removing false positive proteins from the total set of quantified proteins, a global median normalization was carried out to normalize the amount of tryptic digest injected into the nanoLC–MS/MS. Proteins identified in this study are listed in Additional file 2. To select the proteins that showed significant fold-change under the co-culture condition as compared with monoculture, an empirical Bayes moderated *t* test was performed and *p* values were adjusted with the Benjamini–Hochberg method to avoid the problem of multiple testing. Volcano plots were generated to visualize differentially produced proteins for co-culture. The criteria of differentially produced proteins used an FDR-adjusted *p* value <0.01 and fold-change of protein ratio (log_2_) >0.5.

### Pathway analysis

An annotation tool, KEGG pathway of DAVID (Huang et al. 2007) (http://david.abcc.ncifcrf.gov/) was used for functional annotation and pathway analysis of the protein sets. The threshold was set to enrichment score >1.6.

## Results

### Mixed and quantitative proteome analysis for *C. albicans*-macrophage interaction

We first assessed the measurement accuracy of mixed and quantitative proteome analysis. The standard sample consisted of all types of samples in this experiment. The standard sample was separated into three aliquots at a ratio of 0.5:1:2 by volume, respectively. After labeling with TMT reagents with different reporters, the three samples were mixed in a single tube and injected into nanoLC–MS/MS. Each peptide had the approximate expected proportional intensity of reporter ions based the mixed ratio (0.5:1:2) (Additional file 3). This assay showed that each peptide could be quantified with high accuracy, even though the peptides were in a mixture derived from two organisms.

Macrophages infected by pathogens produce TNF-α, which is a cytokine involved in inflammation (Garner et al. 1994). The amount of TNF-α released from macrophages into the culture medium was measured by ELISA (Additional file 4). Macrophage and *C. albicans* monocultures were used as the controls. The amount of TNF-α released from macrophages interacting with *C. albicans* was greater than non-interacting controls and increased in a time-dependent manner with the amount of TNF-α increasing after 3 h of interaction. To identify proteins involved in the mechanism used by *C. albicans* to escape from macrophages, and not the proteins that appeared after escape, an early time of interaction (3 h) was selected for the mixed and quantitative proteome analysis of *C. albicans* interacting with macrophages.

**Figure 2:**
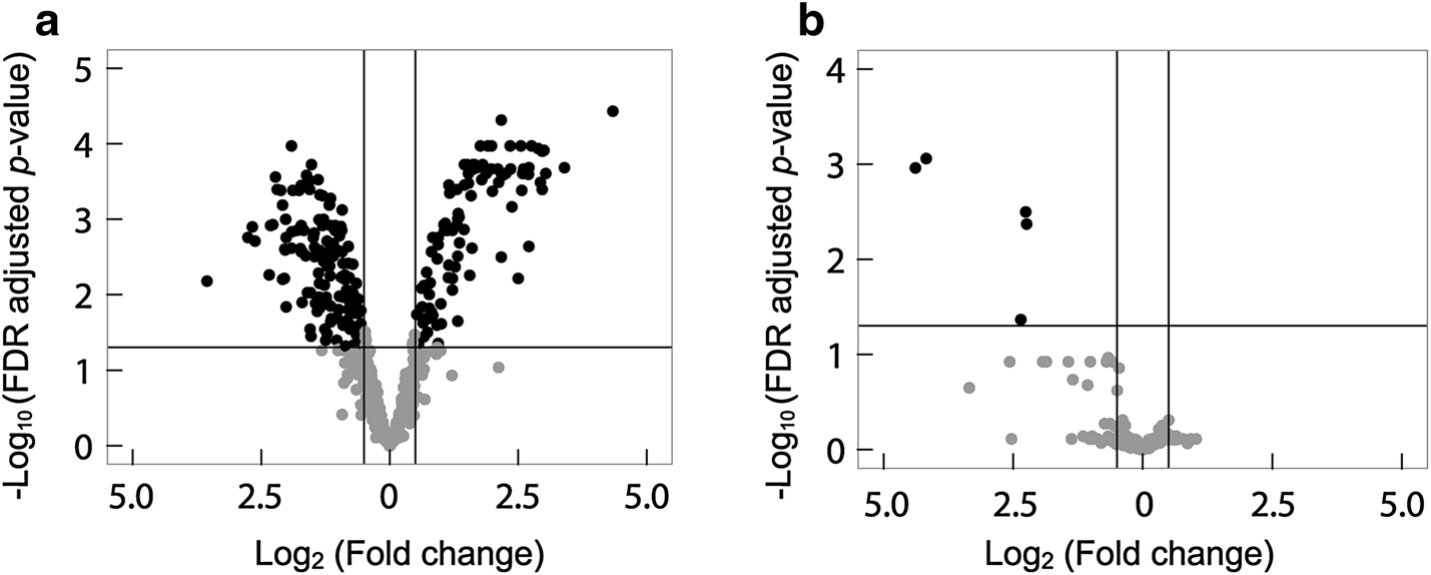
Graphical representations of quantitative proteome analysis data from *C. albicans* (**a**) and macrophage (**b**). Volcano plots were generated by plotting the FDR-adjusted *p*-value against the relative abundance ratio between co-culture and monocultures on a logarithmic scale. The differentially produced proteins were defined as FDR-adjusted *p* value <0.05 and a relative abundance ratio >0.5. Significantly up- (each *right panel*) and down- (each *left panel*) regulated proteins are shown as *black dots* in the volcano plot.

We identified 483 *C. albicans* proteins and 1,253 macrophage proteins using the mixed and quantitative proteome analysis method (Figure 1; Additional file 2). In this analysis, a protein of one organism could be identified in the mixture of peptides derived from two kinds of organisms. Using the *C. albicans* database, 976, 18, and 0 proteins were identified from the *C. albicans* monoculture, the macrophage monoculture, and the complete culture medium, used as background, respectively (Additional files 1, 5). The false-positive rate for *C. albicans* proteins was 1.81% (18 + 0)/(976 + 18 + 0). Using the *M. musculus* database, 21, 1357, and 9 proteins were identified from the *C. albicans* monoculture, macrophage monoculture, and complete culture medium as background, respectively (Additional files 1, 5). The false-positive rate for macrophage protein was 2.16% (21 + 9)/(21 + 1357 + 9). The number of identified proteins was comparable to the number of proteins obtained by conventional proteome analysis that included cell isolation steps (Fernandez-Arenas et al. 2007; Reales-Calderon et al. 2013). This suggests that changes in levels of proteins from the two interacting organisms were simultaneously and efficiently analyzed by our “mixed and quantitative proteome analysis” that used a monolithic column. This analysis method should allow us to identify proteins that are related to the interaction of *C. albicans* with macrophages. To evaluate proteins that showed significant fold-changes between the co-culture and monoculture systems, an empirical Bayes moderated *t* test was performed. Proteins that fulfilled the criteria (FDR adjusted *p* value <0.01 and fold change of protein ratio (log_2_) >0.5) are indicated with black dots in the volcano plot (Figure 2). Ninety-five up-regulated and 132 down-regulated proteins from *C. albicans* (Additional file 6) and five down-regulated proteins from macrophages (Additional file 7) were identified.

### Pathway analysis of *C. albicans* proteins

Proteins that were significantly up- and down-regulated in *C. albicans* (Additional file 6) were grouped according to functions by pathway analysis using KEGG pathway of DAVID (threshold: enrichment score >1.5) (Table 1). We found that 95 of the up-regulated proteins were mainly involved in pathways associated with synthesis of glucose (such as ‘Fatty acid metabolism’ and ‘Glyoxylate and dicarboxylate metabolism’ according to terminology of pathways on KEGG of DAVID), degradation of amino acids (such as ‘Alanine, aspartate, and glutamate metabolism’ and ‘Arginine and proline metabolism’), proteasome function, and stress response (such as ‘Glycerolipid metabolism’). The 132 down-regulated proteins were grouped mainly in ‘Ribosome’. Three conclusions, which will be expanded on below, were suggested by this pathway analysis; (1) with respect to central metabolic pathways, *C. albicans* degrades proteins through proteasomes and generates glucose from the degradation products to prevent glucose starvation inside macrophages; (2) *C. albicans* produces stress-tolerance proteins that help it endure the harsh environment inside macrophages.; (3) *C. albicans* produces candidate pathogenic proteins that allow it to escape from the macrophages.

**Table 1:** Pathway analysis among up- and down- regulated proteins of *C. albicans*

Up-regulated *C. albicans* proteins		Down-regulated *C. albicans* proteins	
Fold enrichment	KEGG pathway term	Fold enrichment	KEGG pathway term
11	Fatty acid metabolism	13	Ribosome
11	Glyoxylate and dicarboxylate metabolism	7.7	Synthesis and degradation of ketone bodies
7.5	Galactose metabolism	5.7	Propanoate metabolism
7.5	Glycerolipid metabolism	5.7	Butanoate metabolism
7.5	Methane metabolism	5.7	beta-Alanine metabolism
7.5	alpha-Linolenic acid metabolism	4.6	Valine, leucine and isoleucine degradation
7.0	Pyruvate metabolism	4.2	Fructose and mannose metabolism
6.4	Citrate cycle (TCA cycle)	3.8	Vitamin B6 metabolism
6.4	Nitrogen metabolism	3.3	Citrate cycle (TCA cycle)
5.6	Butanoate metabolism	3.3	Phosphatidylinositol signaling system
4.5	Pantothenate and CoA biosynthesis	3.0	Glycolysis/Gluconeogenesis
4.1	Fructose and mannose metabolism	2.9	Pyruvate metabolism
3.9	Glycolysis/Gluconeogenesis	2.9	Terpenoid backbone biosynthesis
3.9	Arginine and proline metabolism	2.6	Galactose metabolism
3.9	Alanine, aspartate and glutamate metabolism	2.6	Lysine biosynthesis
3.6	Proteasome	2.3	Amino sugar and nucleotide sugar metabolism
3.4	Amino sugar and nucleotide sugar metabolism	2.3	Lysine degradation
2.8	Propanoate metabolism	2.2	Aminoacyl-tRNA biosynthesis
2.8	Biosynthesis of unsaturated fatty acids	2.1	Glutathione metabolism
2.5	Lysine biosynthesis	1.8	Valine, leucine and isoleucine biosynthesis
2.5	Tryptophan metabolism	1.6	Starch and sucrose metabolism
2.2	Sulfur metabolism	1.5	Purine metabolism
2.0	Glutathione metabolism	1.5	Glycine, serine and threonine metabolism
2.0	Phenylalanine metabolism		
2.0	Oxidative phosphorylation		
2.0	Cysteine and methionine metabolism		
1.9	Phenylalanine, tyrosine and tryptophan biosynthesis		
1.9	Tyrosine metabolism		
1.9	Selenoamino acid metabolism		
1.7	Glycerophospholipid metabolism		
1.7	Valine, leucine and isoleucine metabolism		
1.6	Starch and sucrose metabolism		

### Central metabolic system of *C. albicans*

**Figure 3:**
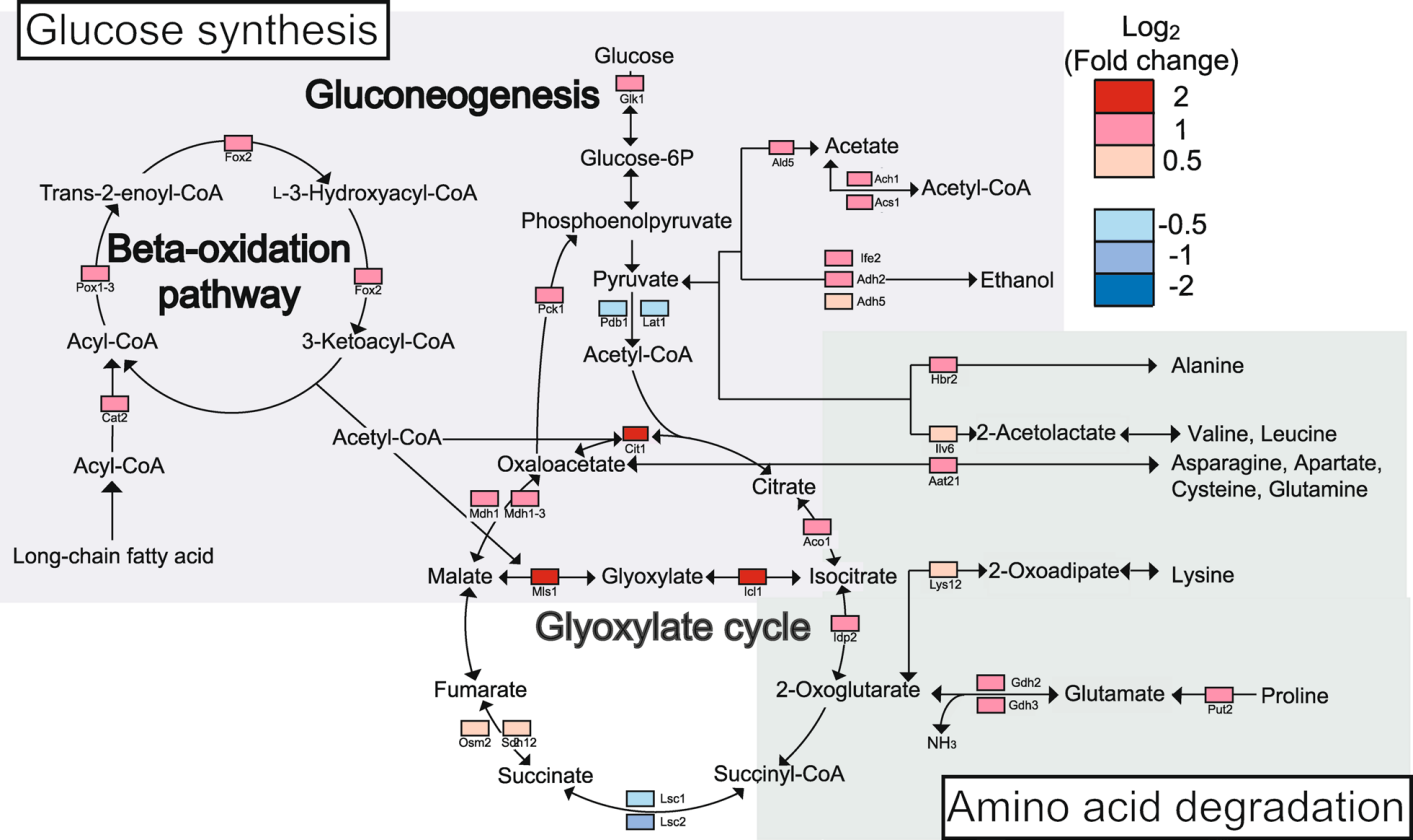
*Candida albicans* proteins for metabolic pathway. *C. albicans* proteins whose syntheses were up- and downregulated are listed in Additional file 6. Here, *squares* denote proteins detected in this study. *Red* and *blue squares* indicate the fold changes of individual proteins.

*Candida albicans* downregulated synthesis of enzymes related to the glycolytic pathway (Lat1, Pdb1) and TCA cycle (Lsc1, Lsc2) (Figure 3). Because there is little free glucose inside the macrophages (Lorenz et al. 2004), it was assumed that *C. albicans* could not generate energy by these pathways. *C. albicans* upregulated enzymes of the fatty acid β-oxidation pathway (Cat2, Pot1-3, Fox2), the glyoxylate cycle (Cit1, Aco1, Icl1, Mls1, Mdh1-3), and gluconeogenesis (Pck1). In a glucose-poor environment, *C. albicans* must produce glucose by these pathways from non-fermentable carbon sources such as fatty acids (Piekarska et al. 2008).

**Figure 4:**
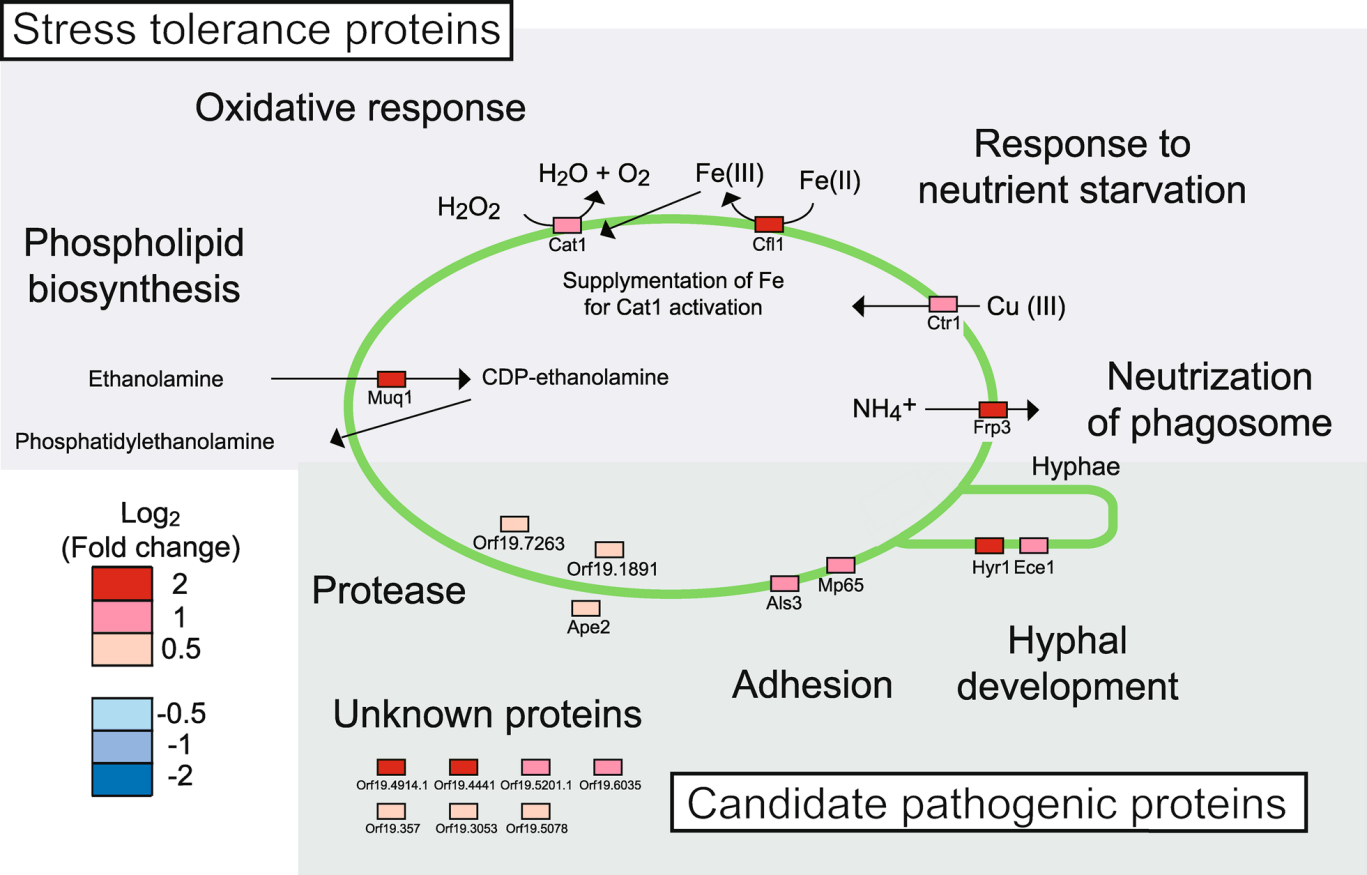
*Candida albicans* proteins for stress tolerance and candidate pathogenic proteins. *C. albicans* proteins whose syntheses were up- and downregulated are listed in Additional file 6. Here, *squares* denote proteins detected in this study. *Red* and *blue squares* indicate the fold changes of individual proteins.

The synthesis of proteins involved with degradation of amino acids was enhanced. Syntheses of enzymes associated with degradation of alanine (Hbr2), valine and leucine (Ilv6), asparagine, aspartate, cysteine, and glutamine (Aat21), lysine (Lys12), glutamate (Gdh2, Gdh3), and proline (Put2) were all upregulated (Figure 3). Syntheses of enzymes related to proteasome function (Pre3, Rpt1, Rpt2, Rpt6, Scl1, Orf19.6582) (Additional file 6) and proteases (Ape2, Orf19.7263, Orf19.1891) (Figure 4) were also upregulated. This suggested that *C. albicans* degraded proteins into amino acids using both proteasomes and proteases.

Nitrogen metabolism also had a high enrichment score in the pathway analysis of upregulated *C. albicans* proteins (Table 1). Of the pathway components, both Gdh2 and Gdh3 (Figure 3) are glutamate dehydrogenases that degrade glutamate to ammonia (Miller and Magasanik 1990). *C. albicans* actively alters the pH of its environment by release of ammonia in vitro (Vylkova et al. 2011). The upregulation of Gdh2- and Gdh3-encoding genes suggested that *C. albicans* used ammonia for pH neutralization inside macrophages. Moreover, the gene encoding the ammonia transporter (Frp3) was also upregulated (Figure 4), and this transporter could contribute to ammonia release (Vylkova and Lorenz 2014).

### *C. albicans* stress-tolerance proteins

In addition to the shortage of glucose and the low pH, *C. albicans* suffers several stresses inside macrophages, including exposure to oxidative stress and a shortage of iron and copper ions (Jimenez-Lopez and Lorenz 2013) (Figure 4).

In our experiments, we found an elevated expression of Muq1-encoding gene, which encodes an enzyme for synthesis of phosphatidylethanolamine (PE), together with a constant amount of Ino1, which is an enzyme associated with synthesis of phosphatidylinositol (PI). These results suggested an overall increase of phospholipid synthesis by *C. albicans* upon interaction with macrophages. Phospholipids could possibly contribute to *C. albicans* pathogenicity.

Macrophages release reactive oxygen species as part of their antimicrobial burst (Vazquez-Torres and Balish 1997). However, *C. albicans* seemed to resist the oxygen species by detoxification using Cat1, which catalyzes the decomposition of hydrogen peroxide to water and oxygen (Miramon et al. 2012). Moreover, synthesis of ferric reductase Cfl1 was upregulated, possibly to allow for capture of iron (III) ions, which are indispensable for Cat1 activity (Hammacott et al. 2000). The other upregulated protein related to resistance to ion shortages was a copper transporter, Ctr1 (Marvin et al. 2003).

### *C. albicans* candidate pathogenic proteins

Many of the upregulated proteins identified in our study have roles in adhesion (Als3, Mp65) (Figure 4). Als3 promotes *C. albicans* invasion into endothelial cells by binding to cadherin and inducing their own endocytosis (Phan et al. 2007). Inside the macrophages, the *C. albicans* adhesion protein might further help with adhesion and escape. Upregulation of some proteases (Ape2, Orf19.7263, Orf19.1891) indicated that proteolysis and utilization of peptides were important for *C. albicans* survival (Figure 4). Some of the upregulated proteins that are related to unknown proteins or hyphal development have not yet been characterized in detail at the functional level (Figure 4). These proteins could be important virulent factors and further studies would be necessary.

### Hypothetical target proteins of *C. albicans* during escape from macrophages

The macrophage proteins whose levels changed during interaction with *C. albicans* are summarized in Additional file 7. Most of the marked proteins were downregulated, not upregulated (Figure 2b). Especially, down-regulation of macrophage apoptosis-associated protein NOA1- and chaperone HSPA1A-syntheses indicated that *C. albican*s was able to escape from macrophages in part by suppressing the production of these macrophage proteins.

## Discussion

Our study has quantitatively characterized the protein profiles during the interaction of *C. albicans* with macrophages using a novel mixed and quantitative proteome analysis system that employs nanoLC–MS/MS with a long monolithic column. We quantitatively identified 483 *C. albicans* proteins and 1,253 macrophage proteins.

Our proteome analysis demonstrated that *C. albicans* upregulated enzymes of the fatty acid β-oxidation pathway, glyoxylate cycle, and gluconeogenesis (Figure 3) To resist glucose-poor environment, *C. albicans* must produce glucose by these pathways from non-fermentable carbon sources (Piekarska et al. 2008). The glyoxylate cycle plays significant roles in *C. albicans*-macrophage interactions, because *C. albicans* mutant lacking *ICL1*-encoding enzyme, which is one of the key enzymes in glyoxylate cycle, was less virulent in mice than wild type (Lorenz and Fink 2001). In addition, independent microarray and qualitative proteome analyses of *C. albicans*-macrophage interactions have suggested the importance of the glyoxylate cycle for *C. albicans* pathogenesis (Fernandez-Arenas et al. 2007; Lorenz et al. 2004). These previous findings are consistent with the results from our mixed and quantitative proteome analyses.

The synthesis of proteins involved with degradation of amino acids and proteasome function, and proteases was enhanced (Figures 3, 4; Additional file 6). This suggested that *C. albicans* degraded proteins into amino acids using both proteasomes and proteases. The resulting amino acids were very likely incorporated into the glyoxylate cycle and gluconeogenesis as 2-oxoglutarate, oxaloacetate, and pyruvate through pathways of amino acid degradation (Rubin-Bejerano et al. 2003). Syntheses of almost all enzymes that have a role only in the TCA cycle (Lsc1, Lsc2, Osm2, Sdh12) were downregulated, but synthesis of isocitrate dehydrogenase was upregulated. The increased amounts of Idp2 (isocitrate dehydrogenase) are probably used for taking 2-oxoglutarate into the glyoxylate cycle (Figure 3).

With respect to *C. albicans* stress-tolerance proteins, we observed high expression levels of enzymes involved in synthesis of PE, which is a constituent of phospholipid (Figure 4). Phospholipids are important components of cell membranes and regulation of their biosynthesis is essential for balanced cell growth (Rattray et al. 1975). The cell membrane plays a crucial role in the virulence of several pathogens. In the fungal pathogen *Cryptococcus neoformans*, Ipc1, which contributes to sphingolipid metabolism, is essential for virulence in a rabbit infection model (Luberto et al. 2001). In the bacterial pathogen *Brucella abortus*, PssA, which is required for PE biosynthesis, is necessary for optimal virulence in a murine model of infection (Bukata et al. 2008). Phospholipids could possibly contribute to *C. albicans* pathogenicity; however, little is currently known about its roles in the pathogenesis of *C. albicans*. One possibility is the restoration of cellular membrane of *C. albicans*, since PE is one of the major components of fungal membrane. PE is a precursor of phosphatidylcholine (PC), which also occupies the major fungal phospholipid (Carman and Han 2011). A *C. albicans* PE synthase mutant, *PSD1*Δ/Δ *PSD2*Δ/Δ is avirulent in a mouse model of systemic candidiasis (Chen et al. 2010), which implies a critical role for PE synthesis. The second possible explanation is the need for synthesis of GPI-anchored proteins. GPI-anchored proteins govern the physiology and pathogenicity of *C. albicans* (Hornbach et al. 2009), and GPI assembly is dependent on PE supply (Orlean and Menon 2007).

**Figure 5:**
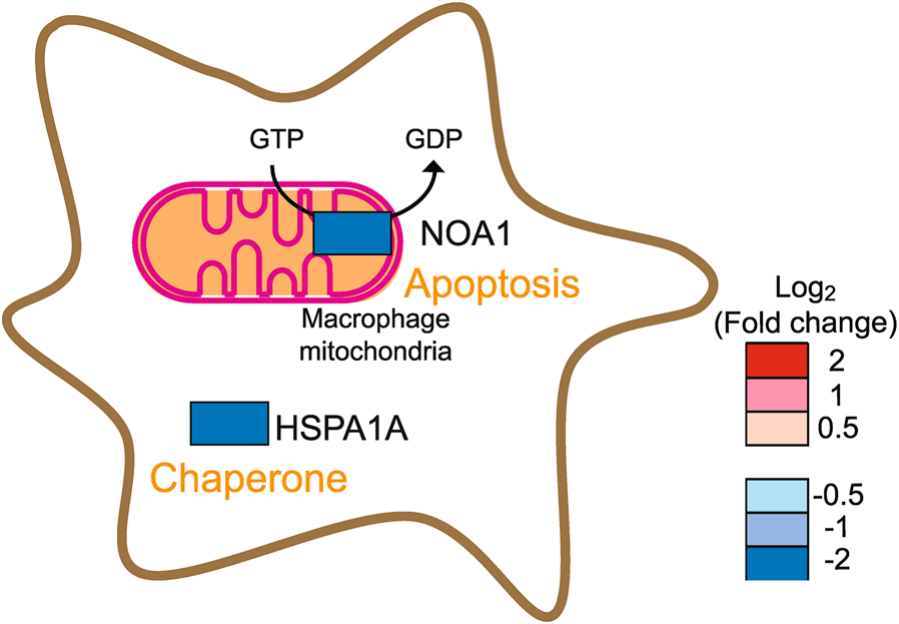
Schematic illustration of macrophage proteins that were differentially regulated. Changes (indicated in *color*) in the macrophage proteins after interaction with *C. albicans* and their putative roles are represented.

Among the differentially produced macrophage proteins that were downregulated, we focused on two proteins, NOA1 and HSPA1A (Figure 5).

NOA1 (Nitric oxide-associated protein 1) is a guanosine triphosphate-binding protein encoded in chromosomal DNA that localizes predominantly in the mitochondrial matrix, and is involved in mitochondrial protein biogenesis and apoptosis. Knockdown of *NOA1* impairs enzymatic activity of the mitochondrial respiratory chain, resulting in oxidative stress and, eventually, apoptosis of C2C12 myoblasts (Heidler et al. 2011). The decrease of NOA1 observed in this study might cause macrophage apoptosis, which could eventually allow for *C. albicans* to escape from the macrophages. Apoptosis has, in fact, been described in macrophages infected with *C. albicans* (Reales-Calderon et al. 2013; Ibata-Ombetta et al. 2003; Gasparoto et al. 2004), although the precise causative mechanisms have not been clarified. Pathogens have evolved diverse strategies to induce host cell apoptosis, which aids in their dissemination within the host (Navarre and Zychlinsky 2000; Santos et al. 2001; Shibayama et al. 2001).

HSPA1A (Heat shock 70 kDa protein 1A) is a chaperone. HSPA1A is assumed to be produced to play a part in the macrophage inflammatory response because transcription of *HSPA1A* is induced by NFκB, a major immune regulator (Sasi et al. 2014). *C. albicans* may lower HSPA1A production to induce macrophage protein instability to help with its escape. Other bacteria, such as *Helicobacter pylori*, have been reported to produce down-modulate host chaperone as part of their immune evasion mechanism (Axsen et al. 2009).

Our results provide novel insights into the relationship of *C. albicans* and macrophage, and should lead to a better understanding of systemic candidiasis and the development of novel drugs.

## Additional files

Additional file 1: False positive proteins of *C. albicans* (18 proteins) and macrophage (30 proteins). Additional file 2: Proteins identified in this study from co-culture of *C. albicans* (483 proteins) and macrophages (1,253 proteins). Additional file 3: Evaluation of protein quantification; The standard sample was combined at a ratio of 0.5:1:2 by volume. After labeling with TMT reagents, the three samples were mixed in a single tube and injected into nonoLC–MS/MS. Relative intensity of reporter ion ratios were obtained by dividing the intensity of reporter ions from the 0.5-sample and the 2-sample by that from the 1-sample. Additional file 4: Levels of TNF-α secreted by macrophages upon interaction with *C. albicans*; Data are represented as mean ± standard deviation (SD) from three independent experiments. Additional file 5: Proteins identified in this study for *C. albicans* monoculture (976 proteins) and macrophage monocultre (1,357 proteins). Additional file 6: Up- (95 proteins) and down- (132 proteins) regulated proteins of *C. albicans* interacting with macrophages. Additional file 7: Changed proteins of macrophage interacting with *C. albicans* (five proteins).

## Abbreviations

*C. albicans*: *Candida albicans*
LC: liquid chromatography
LC–MS/MS: liquid chromatography–tandem mass spectrometry
YPD: yeast extract-peptone-dextrose
FBS: fetal bovine serum
TNF-α: tumor necrosis factor-α
PBS: phosphate buffered saline
TAEB: triethyl ammonium bicarbonate
HCD: high-energy C-trap dissociation
FDR: false discovery rate
