# Supplementary material for: Description of the interaction between Candida albicans and macrophages by mixed and quantitative proteome analysis without isolation
Source: AMB Express. 2015 Jul 16;5:41. doi: 10.1186/s13568-015-0127-2 (PMC4503712; doi:10.1186/s13568-015-0127-2)
Supplement: Additional file 3: — Evaluation of protein quantification; The standard sample was combined at a ratio of 0.5:1:2 by volume. After labeling with TMT reagents, the three samples were mixed in a single tube and injected into nonoLC–MS/MS. Relative intensity of reporter ion ratios were obtained by dividing the intensity of reporter ions from the 0.5-sample and the 2-sample by that from the 1-sample. [file 13568_2015_127_MOESM3_ESM.pdf]

Kitahara *et al*. Additional file 3

article title	Description of the interaction between Candida albicans and macrophages by mixed and quantitative proteome analysis without isolation
journal name	AMB express
author names	Nao Kitahara, Hironobu Morisaka, Wataru Aoki, Yumiko Takeda, Seiji Shibasaki, Kouichi Kuroda, Mitsuyoshi Ueda
affiliation and e-mail address of the corresponding author	Division of Applied Life Sciences, Graduate School of Agriculture, Kyoto University, Sakyo-ku, Kyoto 606-8502, Japan
	miueda@kais.kyoto-u.ac.jp

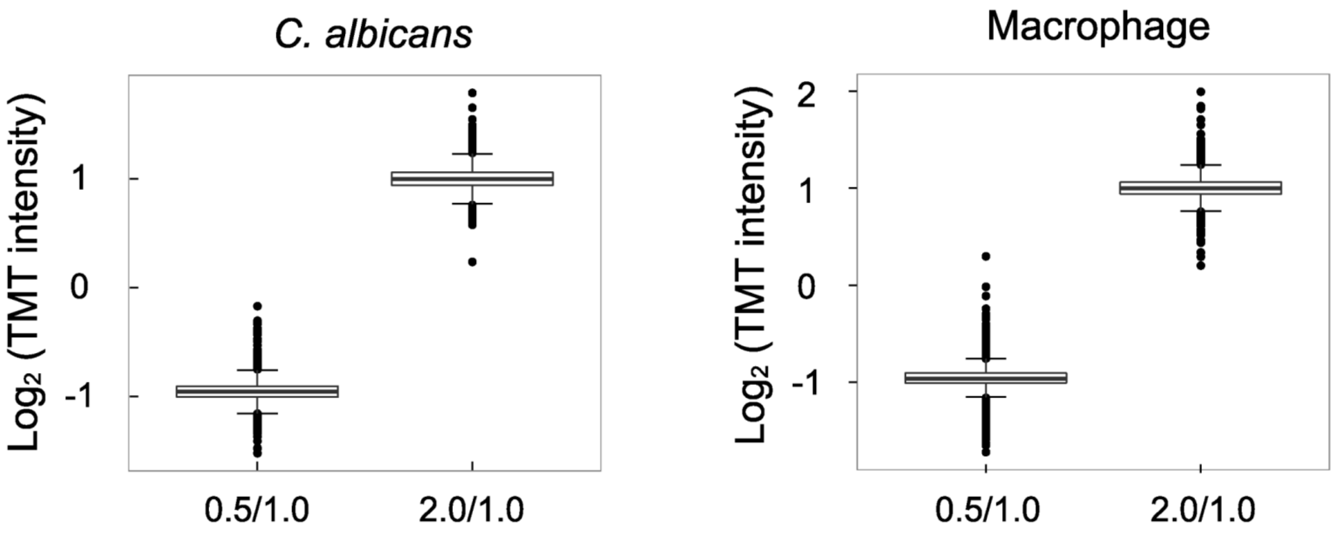

Additional file 3 Evaluation of protein quantification
The standard sample was combined at a ratio of 0.5:1:2 by volume. After labeling with TMT reagents, the three samples were mixed in a single tube and injected into nonoLC-MS/MS. Relative intensity of reporter ion ratios were obtained by dividing the intensity of reporter ions from the 0.5-sample and the 2-sample by that from the 1-sample.
