# Supplementary material for: Description of the interaction between Candida albicans and macrophages by mixed and quantitative proteome analysis without isolation
Source: AMB Express. 2015 Jul 16;5:41. doi: 10.1186/s13568-015-0127-2 (PMC4503712; doi:10.1186/s13568-015-0127-2)
Supplement: Additional file 4: — Levels of TNF-α secreted by macrophages upon interaction with C. albicans; Data are represented as mean ± standard deviation (SD) from three independent experiments. [file 13568_2015_127_MOESM4_ESM.pdf]

Kitahara *et al*. Additional file 4

article title	Description of the interaction between Candida albicans and macrophages by mixed and quantitative proteome analysis without isolation
journal name	AMB express
author names	Nao Kitahara, Hironobu Morisaka, Wataru Aoki, Yumiko Takeda, Seiji Shibasaki, Kouichi Kuroda, Mitsuyoshi Ueda
affiliation and e-mail address of the corresponding author	Division of Applied Life Sciences, Graduate School of Agriculture, Kyoto University, Sakyo-ku, Kyoto 606-8502, Japan
	miueda@kais.kyoto-u.ac.jp

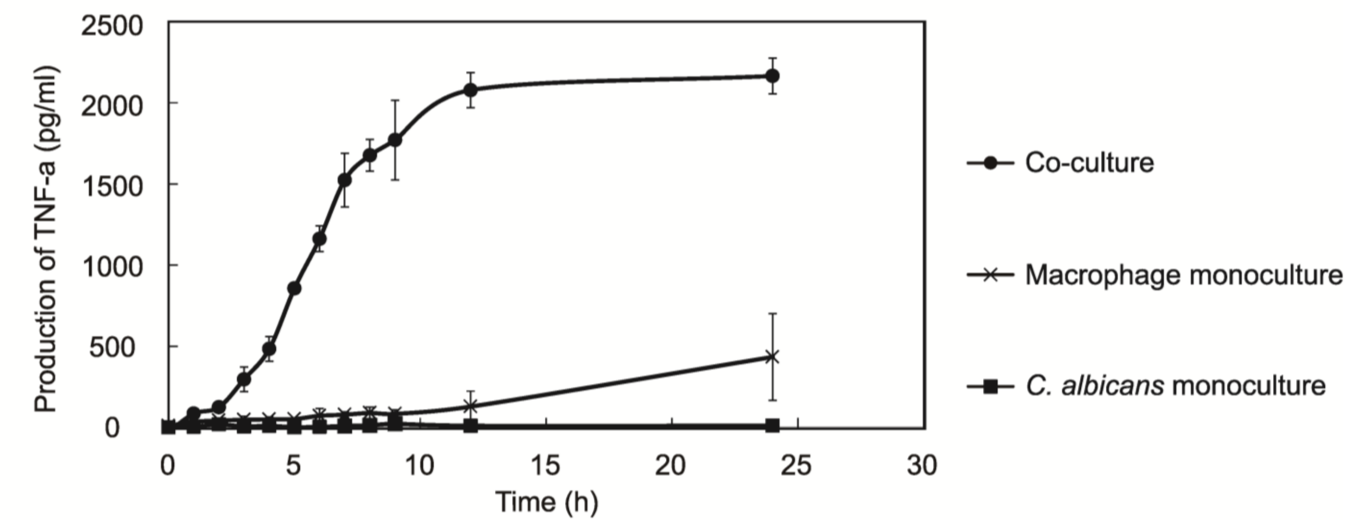

Additional file 4 Levels of TNF-α secreted by macrophages upon interaction with *C. albicans*
Data are represented as mean ± standard deviation (SD) from three independent experiments.
